# Ball-Flight Viewing Duration and Estimates of Passing Height in Baseball

**DOI:** 10.3390/vision9010008

**Published:** 2025-01-25

**Authors:** Emily Benson, Andrew J. Toole, Nick Fogt

**Affiliations:** College of Optometry, The Ohio State University, Columbus, OH 43210, USA; emilybenson.od@gmail.com (E.B.); toole.2@osu.edu (A.J.T.)

**Keywords:** sports vision, ball-flight cues, passing height estimates, situational cues, kinematic cues, cue integration

## Abstract

Predictions of the vertical location of a pitched ball (termed the passing height) when the ball arrives at an observer may be based on internal models of ball trajectory and situational cues, kinematic cues from the pitcher’s motion, and visual ball-flight cues. The informational content of ball-flight cues for judgments of vertical passing height when the ball’s launch angle is small and when situational and kinematic cues are limited is unknown. The purpose of this study was to determine whether passing heights can be judged adequately from ball-flight cues and whether judgments of passing height improve as viewing time increases under the aforementioned conditions. Twenty subjects stood 40 feet (12.19 m) from a pneumatic pitching machine that propelled tennis balls toward them at three speeds (from 53 mph (85 kph) to 77 mph (124 kph)). The ball’s launch angle was constant. The subject’s vision was blocked at 100 ms or 250 ms after pitch release. Subjects indicated the height at which they expected the ball to arrive. In the absence of explicit situational cues or kinematic cues and in the presence of a small and constant launch angle, the longer viewing time of ball-flight cues improved passing height estimates but did not result in accurate responses for the slower speeds.

## 1. Introduction

To successfully avoid or intercept an approaching object, an observer may use multiple sources of trajectory information to determine when and where the object will arrive. This information includes advance cues available prior to the object’s projection, cues from the motion of the object when it is in flight (termed “ball-flight cues” throughout this paper), and predictions of the object’s movement based on prior experience [1,2,3]. In this paper, we focus on a baseball batter hitting a pitched ball. We address the question of whether ball-flight cues presented in the absence of explicit advance cues can be used to assess where an approaching ball will arrive, and whether a longer viewing period improves the reliability of ball-flight cues for these assessments. To answer these questions, we first review the cues available for these judgments.

### 1.1. Cues for Batting

Prior to a baseball pitcher releasing the ball, a number of advance cues for pitch trajectory are available. One group of advance cues are termed contextual cues or situational probabilities. These cues are driven by expectations based on experience or information provided through advanced scouting. For example, a batter may expect a particular pitch type (e.g., fastball or curveball) to be thrown at a specific pitch count (i.e., the number of balls and strikes).

A second group of advance cues to pitch trajectory, termed kinematic cues, are associated with the pitcher’s motion. For example, the pitcher’s arm speed (faster arm speed for a faster pitch) can be considered an advanced kinematic cue. There are also advance non-kinematic cues associated with the pitcher. For example, the appearance of the pitcher’s wrist around the time of ball release (narrower for a slower pitch such as a curve ball) may provide advance information [1,2,3].

Finally, after the pitch is released, there are multiple visual cues available to make judgments of when and where the ball will arrive. These cues include the launch angle of the ball (potentially higher for a slower pitch) as well as other visual cues incorporated into various ratios that may provide information about when and where an object will arrive at the observer. These cues will be explained in the following paragraphs.

### 1.2. Predictions of When a Pitched Ball Will Arrive

The time until an object moving directly toward an observer will arrive at or collide with that individual is termed the time to collision (TTC) or time to contact. Perhaps the most often discussed monocular cue for TTC estimates is the ratio of the instantaneous retinal image size of the object (θ) to the rate of change in the retinal image size (dθ/dt). This ratio is termed tau (Equation (1)) [4,5]. Similar ratios based on binocular disparity have also been derived [6,7].

TTC = θ/(dθ/dt)(1)

Tau is one of a number of potential cues to time to collision [8,9]. For example, Smeets and colleagues suggested that the time to collision could be approximated by dividing the estimated distance of the object by the estimated velocity [10]. Further, Chang and Jazayeri showed that the position of an approaching object relative to other objects at different depths could be used in estimating time to collision [11]. López-Moliner and colleagues developed an equation for time to collision estimates of parabolic trajectories termed the GS (gravity size) model (Equation (2)) [12,13].

(2) $${TTC}_{GS}\approx (2/g)\times (s/\theta )\times (\dot{\gamma }/\cos(\gamma ))$$

where g is gravitational acceleration, s is known ball size, θ is the size of the ball’s retinal image, $\dot{\gamma }$ is the rate of change in the angle of elevation of the ball, and γ is the angle of elevation or launch angle.

While the discussion above is focused on time to collision, time to passage (TTP) is often of interest in athletic endeavors such as baseball batting. TTP is the time remaining until an object that is not on a collision-course with an individual will pass by the observer [14].

### 1.3. Predictions of Where a Pitched Ball Will Arrive

#### 1.3.1. Internal Models

The passing distance (or passing height if assessed from the ground) of an approaching object is the vertical distance of the object from an observer’s eyes when the ball arrives. There are published models describing how an individual might predict an object’s passing distance based on ball-flight cues. Two of these models will be described below, but prior to this, we discuss internal models of object motion.

Through lived experience, it is known that individuals gain an understanding of the influence of gravity on projected objects. Gravity has therefore been described as a “strong prior” [15]. Experiments testing the gravity prior have been performed in virtual environments where the apparent gravitational acceleration can be manipulated [16]. An internal model of projectile motion that includes some combination of gravitational acceleration, an estimate of the time since an approaching ball was released, visual information about the launch angle of the ball, situational probabilities, and kinematic cues might allow an observer to estimate the height upon arrival of the object. Given the limited temporal windows over which a decision as to whether to swing the bat and over which a motor plan for the swing must be formulated (<250 ms), baseball batters may rely heavily on internal models.

Additional forces that act on the ball might be included in this internal model. The Magnus force can alter the motion of a spinning ball as the ball travels through the air. Specifically, the Magnus force occurs due to variations in air pressure around a spinning ball. From a batter’s perspective, the Magnus force may result in less vertical drop if backspin or underspin is imparted to the ball and more vertical drop if topspin or overspin is imparted compared to the case where the ball is not spinning [17]. Gray and Regan demonstrated that individuals were at least indirectly aware of the Magnus force [14]. Another force that acts on a pitched baseball is aerodynamic drag, which is the force from air directed opposite to the direction of the ball’s approach [17]. A stronger drag force could result in the ball dropping to a greater extent than would be expected with a weaker drag force. Presumably, if a baseball batter were to initially formulate a primarily gravity-based internal model to predict the passing distance or passing height of an approaching object, a visual assessment of the rotation of the ball or assumptions about the potential influences of the Magnus force and aerodynamic drag may then be used to modify this original model of ball trajectory.

#### 1.3.2. Ball-Flight Cues

**Figure 1:**
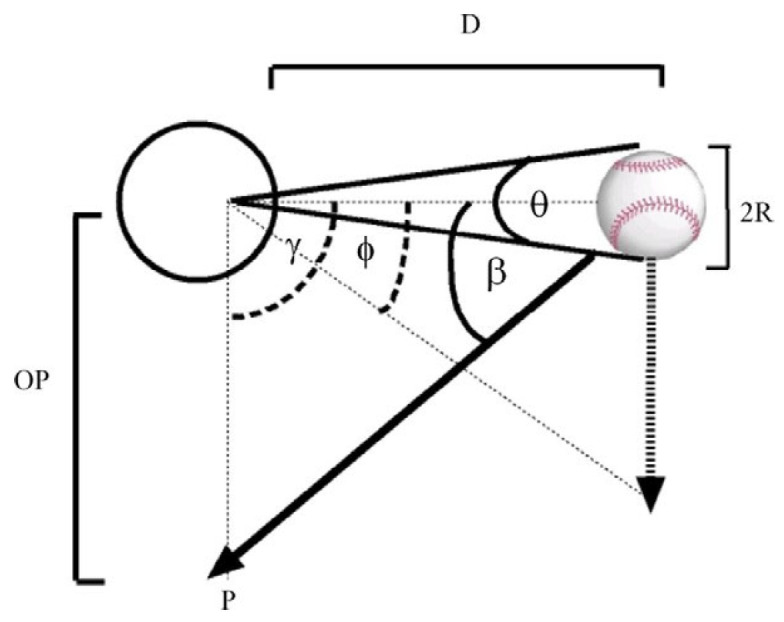
Visual cues to estimate where an approaching object will arrive. Reprinted from *Vision Research*, 46(15), Gray and Regan, Unconfounding the direction of motion in depth, time to passage and rotation rate of an approaching object, 2388–2402, Copyright 2006, with permission from Elsevier [14]. Angle ϕ is the visual direction of the approaching ball, angle β is the ball’s approach angle, R is the ball’s radius, D is the instantaneous distance of the ball from the observer’s eye (open circle), θ is the instantaneous angular subtense of the ball, γ is the optical angle at the eye between the location of the ball and point P, and OP is the passing distance.

In addition to an internal model of projectile motion, specific models applying ball-flight cues to estimate where an approaching object will arrive have been proposed. These models will be described here, because they include those cues that observers may use in estimating the passing height of approaching objects. Bahill and Karnavas published the equation below in the context of baseball batting [18].

(3) $$\hat{z}=({\hat{d}}_{0}-{\hat{t}}_{\mathrm{SR}}\times \hat{s})\times ((d\phi /dt)\times (\theta /(d\theta /dt))$$

where $\hat{z}$ is an estimate of the vertical distance of the ball from the eye of the batter at the front of the plate, ${\hat{d}}_{0}$ is the distance from the batter to the pitcher, ${\hat{t}}_{\mathrm{SR}}$ is the elapsed time since the ball was released by the pitcher, $\hat{s}$ is the linear ball speed, dϕ/dt is the angular drop speed of the ball as determined from the ball’s retinal image velocity, and θ/(dθ/dt) is tau and represents the time to collision. The values of dϕ/dt and θ/(dθ/dt) are available in the retinal image. However, both the time since the ball was released (${\hat{t}}_{\mathrm{SR}}$) and the speed of the ball $\hat{s}$ must be estimated to determine the current distance of the ball from the plate. Once the distance is estimated, then it is possible to obtain the linear drop speed by combining the angular drop speed and the estimated distance of the ball. Gray and Regan [14] proposed a model of passing distance estimates similar to that of Bootsma and Peper [19] that utilizes the estimated or known size of an approaching ball. This model is based on the equation below (symbols from Gray and Regan) (Figure 1):

OP ≈ (2R(dϕ/dt))/(dθ/dt)(4)

where OP is the vertical distance from the observer’s eye when an approaching object passes the observer, R is the radius of the object, dϕ/dt is the vertical angular drop rate or vertical velocity of the object, and dθ/dt is the rate of change in the retinal image size [14].

Important variables in the models of Bahill and Karnavas [18] and Gray and Regan [14] might be suprathreshold relatively early in the pitch. The rate of change in the angular size of the ball (the denominator of the tau ratio) is thought to be over the detection threshold (0.08 deg/s or less) as soon as the pitch is released. At least for motion in the frontal plane, McKee demonstrated that relative differences in retinal image velocity of less than 5% (Weber fraction) can be discriminated over a large velocity range of 2–64 deg/s [18,20,21]. Sekuler reported a similar Weber fraction for the discrimination of the speeds of approaching or looming stimuli ($\hat{s}$ in Equation (3) above) [22]. The angular retinal image size of a looming stimulus increases as it moves toward an observer. However, in contrast to the results of Sekuler, Rushton and Duke demonstrated that speed discrimination for a flying object as it approaches is inaccurate [23]. In the current experiment, cues for estimates of linear speed or at least estimates of relative differences in linear speed are plentiful. Potential cues include the distance traversed over a given exposure duration (because the distance traversed during the time the ball is visible increases with increasing speed for a given exposure duration), looming cues, binocular cues (increase in retinal disparity as the object approaches and interocular velocity differences between the eyes) [24], and changes in the angle between the observer and the approaching object.

One variable that could influence speed discrimination and that is related to the current experiment is the stimulus exposure duration. McKee’s study, using stimuli moving in a frontal plane, showed that the Weber fraction of less than 5% for retinal image velocity discrimination held for stimulus durations of 200 ms. De Bruyn and Orban, using random dot stimuli moving in a frontal plane, showed that velocity discrimination improved as exposure duration increased up to about 150–200 ms (depending on the stimulus velocity) [25]. We are not aware of studies assessing the influence of the very short (≤250 ms) exposure durations used in the current experiment on the discrimination of speeds for approaching (i.e., looming) stimuli.

Generally, studies have shown that ball-flight cues are integrated with contextual and kinematic cues [26]. Reliance on a particular cue is dependent on the reliability of the cue at the time in the trajectory at which these cues are examined. For example, Gray and Cañal-Bruland showed that baseball batting performance was improved when a combination of information sources was available [27]. That is, performance was best if batters knew the most likely pitch type to be thrown and if batters saw the first 150 ms of the pitch trajectory as opposed to less than 150 ms of the initial pitch trajectory. If both the pitch type was known and batters saw the first 150 ms of the pitch trajectory, then the early portions of the bat swing were best correlated with the known pitch type while the later portion of the swing was correlated with the visual information from the early (150 ms) portion of the ball’s flight. Finally, Gray and Cañal-Bruland concluded that batting efficiency and swing timing were influenced to a greater extent by the probability of a particular pitch type when the amount of time available to assess ball-flight cues was less than 150 ms.

In a virtual reality setting, Nakamoto and colleagues asked baseball players which of two pitched balls had the greater velocity in a scenario where the ball speeds were constant but the pitcher’s motion was varied [28]. The pitcher’s motion influenced the apparent ball speed, and this influence was greater at faster ball speeds. The authors attributed the greater effect of the pitcher’s motion on perceived ball speed at the faster ball speeds to less reliable ball-flight information when the pitch was faster. de La Malla and López-Moliner examined performance in a virtual catching task. Subjects were required to “catch” an approaching ball with different parabolic trajectories [29]. The ball was visible either early in the trajectory, or late in the trajectory, or for nearly all of the trajectory. These investigators concluded that early ball-flight information related to the ball’s launch angle could be integrated with late ball-flight trajectory information (e.g., rate of ball expansion). Specifically, these investigators demonstrated that when only the early portion of an object’s trajectory is seen, early sources of information including the known size of the approaching object, knowledge of gravitational acceleration, the angle of elevation of the projected object, and the optical or visual angular size of the object are particularly useful for time to collision estimates for catching. On the other hand, later in the object’s approach, these investigators suggested that information contained in an equation that includes the rate of expansion of the object’s retinal image becomes more significant in establishing the time to collision.

Brantley and Körding have applied Bayesian statistics to baseball batting [30]. Specifically, these investigators attribute batters’ decisions about whether and where to swing the bat to combinations of two probability distributions. The first distribution, termed the prior, includes potential locations where a pitched ball may arrive based on advance cues. This distribution therefore contains elements of the internal models described above. The second distribution, termed the likelihood function, includes potential locations at which the pitch may arrive based on information around the time the pitcher releases the ball (e.g., the location of the pitcher’s arm upon release of the ball, the pitcher’s grip on the ball, and the spin of the ball) and based on early ball-flight information. Information from these two distributions is combined to formulate a posterior probability of locations at which the ball is likely to arrive. The extent to which each of the probability distributions (the prior and likelihood functions) is relied upon depends on the relative certainty of the information (that is, the amount of noise) in each of these distributions. For example, if a longer period of time is allowed to assess ball-flight cues, which would usually be the case with slower pitches, then the likelihood function might have a greater influence on the posterior probability of the pitch arriving at a particular location. While Bayesian analysis and the studies of cue integration described above suggest that the fidelity of ball-flight cues for estimating time to collision and perceived ball speed may improve over time, a major question remains.

This question is whether ball-flight cues can be used independently of explicit contextual cues and kinematic cues (that is, information associated with the prior distribution in Bayesian terms) to predict the passing height of an approaching ball when the launch angle is minimized and kept constant at different ball speeds. It is possible that ball-flight cues in the absence of other cues are unreliable (or considered by observers to be unreliable), and that the reliability of these cues does not improve as the exposure time of the approaching ball increases. This could be the case if ball-flight cues primarily serve to refine an already existing but potentially coarse internal trajectory model. In a study of passing height estimates with very short viewing durations (about 100 ms), Fogt and Sander suggested that in the absence of strong (and relatively reliable) contributions to this internal model from situational probabilities and kinematic cues, passing height estimates might be based on educated guesses derived from previous experience [31].

The first purpose of this study was to determine whether an internal model that includes educated guesses based on experience in batting baseballs dominates over information from ball-flight cues for passing height estimates of approaching balls when the ball’s launch angle is small and constant for different ball speeds and when situational cues and kinematic cues are limited. The second purpose was to assess whether information from ball-flight cues is more viable or more relied upon for passing height estimates later in the pitch trajectory. Multiple ball speeds were used because previous results suggested that limiting the exposure duration of the pitches would have a differential effect on judgments of passing height at different speeds [31], because models of passing height judgments suggest that assessing the approach speed is important [18], and because batters in baseball and softball often face pitches thrown at different speeds in a single at-bat [2]. The results give insight into whether ball-flight cues can be evaluated and used for passing height estimates independently of an internal model of trajectory built on the gravity prior and influenced by situational and kinematic cues, or whether ball-flight cues in isolation are not sufficient for passing height estimates.

## 2. Materials and Methods

### 2.1. Subjects

This study and its associated forms were approved by the Ohio State University Biomedical Institutional Review Board. In total, 20 adults participated (12 males, 8 females; mean age 23.7 ± 1.69, age range 20 to 27). Subjects were required to have 20/20 best corrected monocular visual acuity in each eye, no strabismus with a unilateral cover test in primary, right, or left gaze, and a stereoacuity of at least 60 s of arc (Randot). None of the recruited subjects failed this pre-screening. Subjects were also required to have played organized baseball or softball at the high school level or above within the past 10 years. Prior to any testing, each participant signed a written informed consent document and completed a survey on baseball experience to ensure that they qualified for the study.

### 2.2. Equipment

This study was performed in an indoor laboratory at the Ohio State University. Tennis balls were “thrown” using a compressed air pitching machine (Flamethrower, Accelerated Baseball Technologies, Crystal Lake, IL, USA). The balls were placed into the pitching machine one at a time. The balls exited the pitching machine through an attached PVC tube at a height of 5.1 feet (1.55 m) above the ground. The tube had a series of drilled holes to allow for ball speed adjustment. A black curtain was used to obscure the speed adjustment mechanism from the subjects. The tube was nearly parallel to the ground, such that the launch angle of the balls was less than about 2 degrees.

An infrared rectangular ballistic timing window (Model 57 Photoelectric Screen, Oehler Research, Austin, TX, USA) was placed at the end of the pitching machine tube. When a ball passed through the timing window, the analog output from this window varied and this change in voltage indicated the time at which the ball was released from the pitching machine tube. The voltage from the timing window was fed into an 11-bit analog-to-digital converter (MCC USB-1208FS, Digilent, Pullman, WA, USA) and sampled at 1000 Hz.

**Figure 2:**
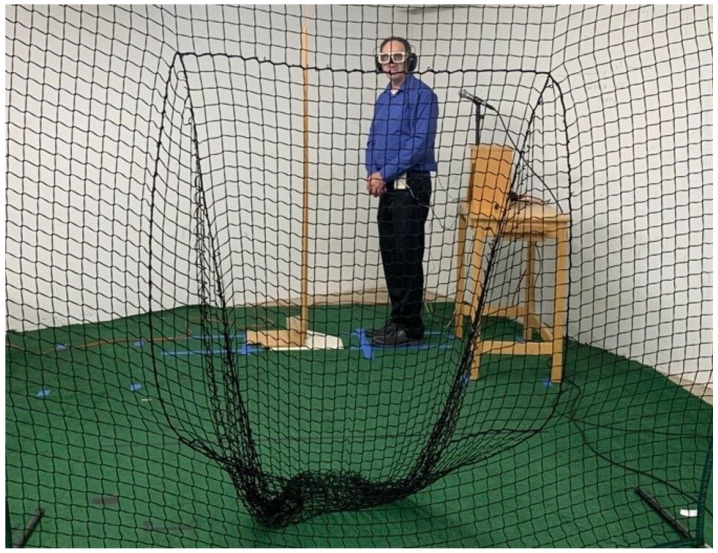
One of the investigators standing in the location occupied by the subjects and wearing the occclusion spectacles and headphones. The investigator is looking in the direction of the pitching machine. Participants stood adjacent to a (white) plate and viewed a 2 m ruler placed on the plate as shown. The net is shown in front of the plate. Informed consent was obtained from the person pictured in this figure.

During the experiment, a net was placed about 8 feet (2.44 m) in front of the subject to stop the balls before they reached the subject (Figure 2). The subjects stood next to a plate placed on the ground 40 feet (12.19 m) from the end of the pitching machine. They stood within a “box” marked on the ground as if they were going to bat the balls thrown by the pitching machine. As the ball approached the net, it passed two vertical structural columns at different distances that extended from the floor to the ceiling.

Participants were given a pair of noise-canceling headphones that played white noise to limit auditory cues associated with both the release of the ball from the pitching machine and with the ball striking the net. Subjects also wore Plato occlusion spectacles (Translucent Technologies, Toronto, ON). The opening (and closing) of these spectacles was controlled by the same analog-to-digital converter that was used to monitor the voltage from the ballistic timing window at the end of the pitching machine tube. A digital signal from the analog-to-digital converter was used to open (and close) the occluding spectacles. When the occluding spectacles were “closed”, the spectacles scattered light.

The subject’s task was to indicate the height (to the nearest centimeter) above the ground that they expected the ball to arrive by referring to a vertically oriented 2 m ruler placed on the plate next to the subject (Figure 2). The subjects were instructed to lean down in order to read the height on the ruler. Most subjects placed their hand at the point where they expected the ball to pass and read the number nearest to their hand from a mostly vertical stance. Some subjects simply peered at the ruler and then stated their prediction. The number of subjects who placed their hand at the expected passing height was not recorded, and the physical height of each subject was not assessed.

Three pitch speeds were used in the experiment. The average speeds were about 77 mph (124 kph) (speed 1), 61 mph (98 kph) (speed 2), and 53 mph (85 kph) (speed 3). These speeds were determined in a separate session using the timing window at the end of the pitching machine tube and a timing window consisting of photodiodes that was built by the experimenters and placed at the front or leading edge of the plate. The average and standard deviation (20 pitches) of the times required to traverse the 40-foot distance (the entire distance from the pitching machine to the plate) are shown in Table 1.

In addition to the just-described timing measurements, the measured height (hereafter referred to as the expected height) upon arrival of the balls at each speed was also assessed (Table 1). To obtain these expected heights, 30 (high and medium speeds) or 31 (low speed) pitched balls were projected by the pitching machine at foam poster boards which were mounted vertically on a wooden board and placed at the leading edge of the plate. When the balls struck the poster boards, an indentation remained at the location where the ball contacted the board, and therefore the height of the ball could be determined. The expected heights were taken as the midway point between the highest and lowest vertical locations at which the ball arrived. The range of expected heights was the difference in height between these extreme vertical locations.

### 2.3. Experimental Protocol

Once subjects were positioned adjacent to the plate, they were shown two “sample” pitches. One of these pitches was thrown at the highest speed used in the experiment (77 mph) and the other was thrown at the lowest speed (53 mph). The order of these pitches was randomized for each subject by a coin flip. Subjects were permitted to watch these pitches until they struck a net placed about 4 feet (1.22 m) in front of the plate. Subjects were informed that these two pitch speeds might be used in the experiment, but no information was given regarding the speed of these pitches or how many ball speeds would be utilized in the experiment. The sample pitches were shown because a previous study demonstrated that subjects consistently overestimated the predicted height of pitched balls at an exposure duration of 100 ms [31]. It was thought that if subjects were aware that pitches could potentially arrive at very low locations, this could positively influence their estimates of ball height by providing additional advance or prior information.

**Figure 3:**
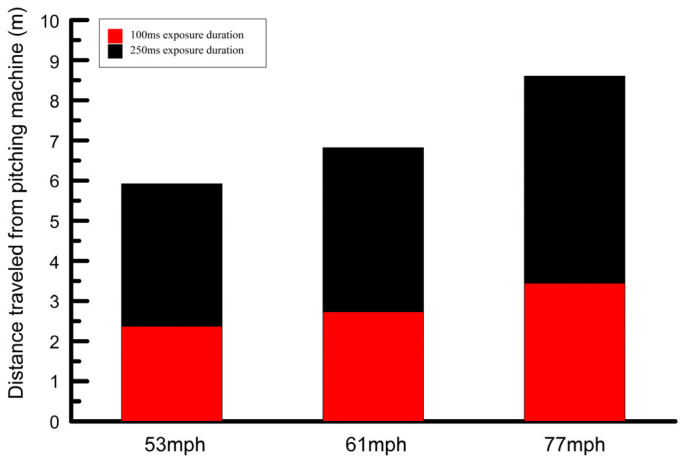
The distance from the pitching machine that the ball traveled prior to occlusion for the 100 ms (red) and 250 ms (black) exposure durations. The total distance from the pitching machine to the plate was 12.19 m (40 ft).

Next, the data collection portion of the experiment commenced. The net in front of the subject was placed about 8 feet (2.44 m) in front of the subject. A computer program written by one of the investigators was used to control all aspects of the experiment. The computer program would indicate to the investigators the speed of the pitch to be thrown. An experimenter would adjust the sleeve on the pitching machine tube accordingly and drop a ball into the pitching machine. The subject’s vision was then occluded by the occluding spectacles at 100 ms or 250 ms after the ball left the pitching machine tube as determined from the timing window. The constant exposure durations resulted in a potential distance cue for the relative speeds, in that for a given exposure duration, the ball traveled furthest for the fastest speed prior to occlusion (Figure 3). Following a random time delay after each pitch struck the net, the occluding spectacles were “opened” and the subject was asked to verbally indicate the height above the ground that the ball would have arrived had it reached the plate. The subjects were therefore required to extrapolate the ball’s motion. The subjects were given unlimited time for their responses. These responses were recorded in the computer program.

For each subject, data were recorded for 60 pitches. The computer program randomly selected the speed of the pitch and the exposure duration. Each speed was used 20 times, and each exposure duration was used 10 times at each speed. Therefore, 6 combinations of speed and exposure duration (3 speeds × 2 durations) were used in the experiment. For eight subjects, there were 1 or 2 pitches for which the subject felt that a response could not be given. Subjects were not asked to explain why a response could not be given, but potential reasons could be a shift in gaze away from the ball’s path or a lapse of attention. For these latter cases, at some point during the trial, those pitches were shown again. There was one trial for one subject where the occluding spectacles failed to close and at some time during the trial that pitch was used again.

### 2.4. Data Analyses

A two-factor repeated measures analysis of variance was performed in IBM SPSS version 28 (IBM Corporation, New York, NY, USA). The outcome measures were the passing height responses and the factors in the model were ball speed and exposure duration. A second two-factor repeated measures analysis of variance was also performed using the standard deviation of the mean passing height responses for each subject as the outcome measures. The factors in the model were once again ball speed and exposure duration.

Paired *t*-tests, performed in Minitab version 21 (Minitab LLC, State College, PA, USA), were used to compare the height responses for the two exposure durations at each speed. These tests were performed because the results of the repeated measures analysis of variance showed a significant interaction for ball speed and exposure duration. Similarly, paired *t*-tests were also performed to compare the standard deviations for the two exposure durations at each speed. Bonferroni correction was used to account for multiple comparisons for all paired *t*-tests (*p* < 0.017). The accuracy of the passing height responses for each combination of speed and exposure duration was assessed in two ways. First, the mean responses were compared to the expected height responses (middle of the measured range of heights) using one-sample *t*-tests. Second, the accuracy of the passing height responses was assessed by determining the percentage of responses that fell in the expected (i.e., measured) range at each combination of speed and exposure duration.

For each subject, the mean differences between each subject’s passing height responses and the expected heights (middle of the range of measured heights) were also determined, as were the standard deviations of these means.

Two important variables for the judgment of passing height, the rate of retinal expansion and the vertical retinal image velocities, which were included in the equations described in the introduction [14,18], were assessed to determine if these variables exceeded the discrimination threshold for the three velocities at the two exposure durations. Rates of retinal expansion were determined at exposure durations of about 100 ms and about 250 ms for the three stimulus velocities by calculating the rate of change in the angular subtense of the ball’s retinal image over a time interval of 10 ms (5 ms prior to the exposure durations used in the experiment to 5 ms after these exposure durations). The distance of the ball used to obtain the angular subtense at each time of interest in the trajectory was determined assuming a constant linear ball speed. The rate of retinal expansion ranged from about 1 deg/s to about 10 deg/s.

**Figure 4:**
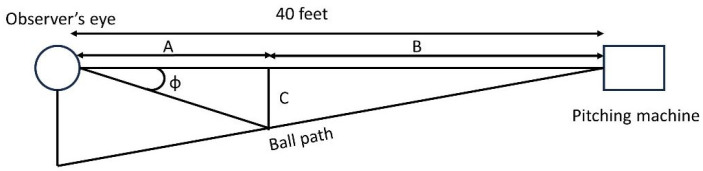
Diagram associated with retinal image velocity calculations. The line marked “Ball path” will follow a parabolic path. A is the (instantaneous) distance of the ball from the eye, B is the distance the ball has traveled from the pitching machine, C is the vertical distance traveled by the ball, and ϕ is the angle between a line parallel to the eye and a line from the eye to the ball.

Vertical retinal image velocities were also calculated at elapsed times in the pitch trajectory of about 100 ms and 250 ms. The retinal image velocity at each combination of speed and exposure duration was calculated as follows. Referring to Figure 4 below, if one assumes a constant ball speed, then at a particular time (t) after the ball is released, the distance the ball has traveled (distance B in Figure 4) is equal to the ball’s speed multiplied by the elapsed time since the pitch was released (t). Subtracting distance B from the total distance from the pitching machine to the observer’s eye results in distance A, which is the distance from the eye to the ball at time t. The vertical distance that the ball has dropped is C and can be calculated using the equation −1/2 gt^2^ where g is the gravitational acceleration (9.8 m/s^2^). The angle (of the ball) between a line parallel to the eye and a line from the eye to the location of the ball can be calculated as tan ϕ = C/A. Calculating the difference between angle ϕ at two elapsed times at or near the elapsed time of interest (5 ms prior to the exposure durations used in the experiment to 5 ms after these exposure durations) and dividing the difference in these angles by the difference in elapsed time (10 ms) associated with these two angles yields the retinal image velocity at or near the elapsed time of interest.

## 3. Results

### 3.1. Mean Passing Height Responses

From the survey responses, all of the female subjects responded that they played softball while all of the male subjects responded that they played baseball. Seven subjects responded that they were a batter, while the other thirteen subjects answered that they were both a pitcher and a batter. In terms of the highest level that subjects competed in, fifteen subjects answered “high school”, four “college”, and one “varsity”. It was unclear whether “varsity” referred to high school or college.

**Figure 5:**
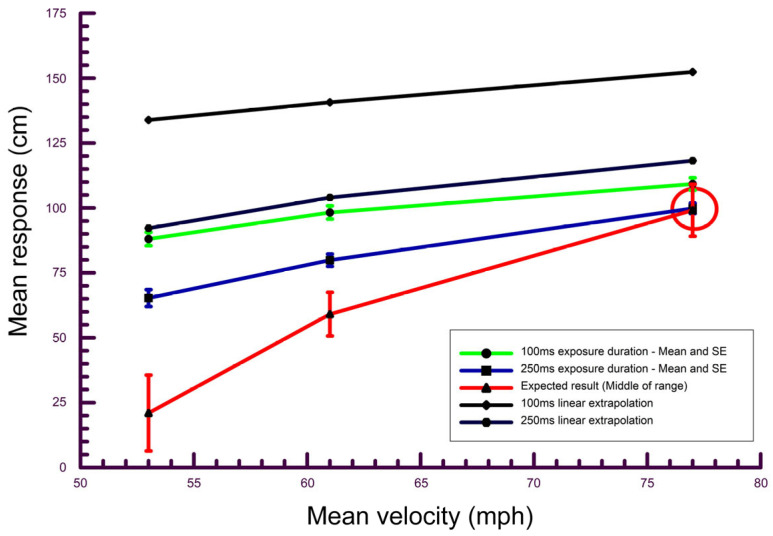
Mean height responses (mean of subject means) and the middle of the range of expected responses at each ball speed (green = 100 ms exposure duration, blue = 250 ms exposure duration). Error bars are plus/minus the standard error for the height responses, and the measured range (from highest measured ball height to lowest measured ball height) for the expected responses. The circle indicates the only scenario (high speed, 250 ms viewing duration) at which the mean response was not significantly different from the expected response. The upper black line is the calculated height responses if subjects applied a linear extrapolation from visual information available at the 100 ms exposure duration and the lower black line is the calculated height response if subjects applied a linear extrapolation from visual information available at the 250 ms exposure duration.

**Figure 6:**
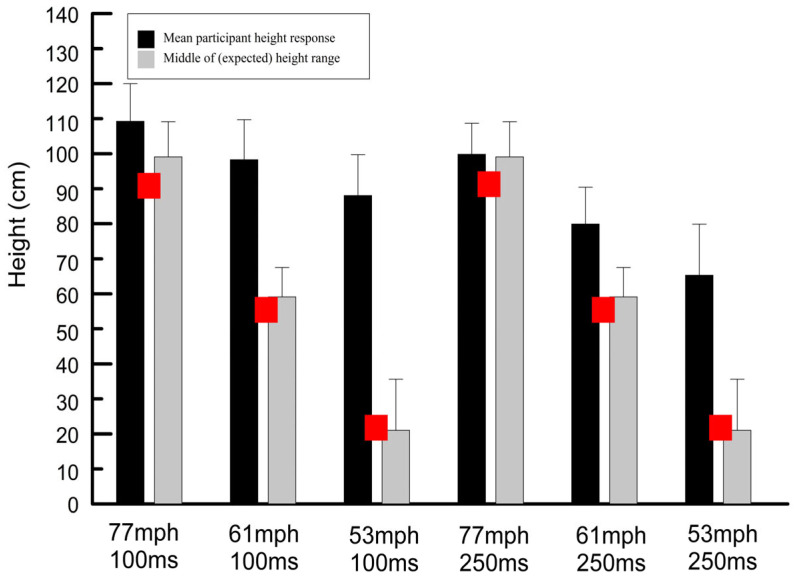
Mean height responses (black bars) and standard deviations compared to the middle of the range of expected responses (gray bars) for all combinations of ball speed and exposure duration. The center of the red squares represents the calculated heights based on the equation −1/2 gt^2^.

The mean height responses for each subject at each speed and viewing duration combination were calculated (Appendix A, Table A1), as were the overall means (mean of subject means). The overall means are shown in Table 1 and Figure 5 and Figure 6. Included in this table and the figures is the range of measured ball heights (red in color and labeled “expected height” in Figure 5 and gray bars in Figure 6). The calculated height of the ball assuming a launch angle of zero and assuming no Magnus force or drag is also shown in Table 1 and Figure 6. These latter values were calculated using the equation −1/2 gt^2^ where t is the measured time to traverse the 40-foot (12.19 m) distance from the pitching machine to the plate [32]. Finally, the measured times required for the ball to traverse the 40-foot distance from the pitching machine to the plate are included in Table 1.

Included in Figure 5 are two black lines showing the expected height responses based on a linear extrapolation at the two exposure durations. These values were calculated to assess whether subjects may have applied such an extrapolation for their passing height estimates. The extrapolation to the plate was made by first measuring the ball height when the ball struck a foam board at distances where the ball was expected to be after 100 ms and 250 ms. These latter distances were calculated assuming a constant linear ball speed. After that, the ratios of the change in ball height from the pitching machine to the distances (from the subject) at which the ball was expected to arrive after 100 ms and after 250 ms were calculated. Finally, these just-described ratios were used to determine the height of the balls at the plate from this linear extrapolation.

The results of the two-factor repeated measures analysis of variance for the passing height estimates were as follows. The interaction term (exposure duration × ball speed) was significant (DF 2, 38, *p* < 0.001, $\eta_{p}^{2}$ = 0.479), and ball speed (DF 2, 38, *p* < 0.001, $\eta_{p}^{2}$ = 0.894) and exposure duration (DF 1, 19, *p* < 0.001, $\eta_{p}^{2}$ = 0.850) were also significant.

All of the paired *t*-tests comparing the passing height responses for the two exposure durations at each speed were statistically significant (*p* < 0.001), and in all cases the passing height response for the longer exposure duration (250 ms) was lower and therefore better than that for the shorter exposure duration (100 ms) as shown in Figure 5 and Figure 6 and Table 1. These data suggest that longer exposure durations lead to better mean passing height estimates.

### 3.2. Accuracy of Mean Passing Heights

The accuracy of the passing height responses for each combination of speed and exposure duration were assessed in two ways. First, the mean responses were compared to the expected passing height responses (middle of the measured range of heights) using one-sample *t*-tests. All of these comparisons indicated that the mean responses were significantly different from the expected value (*p* < 0.001) except for the combination of the highest speed and longer (250 ms) exposure duration (*p* = 0.714). Second, the percentage of responses that fell in the expected (i.e., measured) range at each combination of speed and exposure duration were calculated (Table 2).

Paired *t*-tests of the mean percent correct for the two exposure durations were compared at each speed. The differences in percent correct were significantly different at the highest speed (*p* = 0.001) and the middle speed (*p* < 0.001). In both of these latter cases, the percent correct was greater for the longer (250 ms) exposure duration, reflecting better performance. However, at the lowest speed, the values for the two exposure durations were not significantly different (*p* = 0.163). The accuracy of the height responses as assessed by the percent correct metric suggests that performance was relatively poor in all cases, but it was better for the longer (250 ms) exposure duration than the shorter (100 ms) exposure duration at the high and medium ball speeds.

### 3.3. Mean Variability for Passing Height Responses

A two-factor repeated measures analysis of variance was also performed for the variability (standard deviation of the means) for the passing height responses. It was thought that a longer exposure duration may result in reduced variability of the responses, particularly if subjects were relying on potentially unreliable ball-flight cues. The Greenhouse–Geisser correction was applied. Ball speed was a significant factor (DF 2, 38, *p* = 0.004, $\eta_{p}^{2}$ = 0.255) and the interaction term was significant (DF 2, 38, *p* = 0.006, $\eta_{p}^{2}$ = 0.248). The mean standard deviations were greater at the two slower ball speeds compared to the fastest ball speed. The increased variability at slower ball speeds may be partially attributable to the larger range of measured ball heights at the slower speeds. Exposure duration was not significant (DF 1, 19, *p* = 0.847, $\eta_{p}^{2}$ = 0.002), suggesting that exposure duration did not affect the variability of the height responses.

### 3.4. Mean Constant and Variable Passing Height Errors

**Figure 7:**
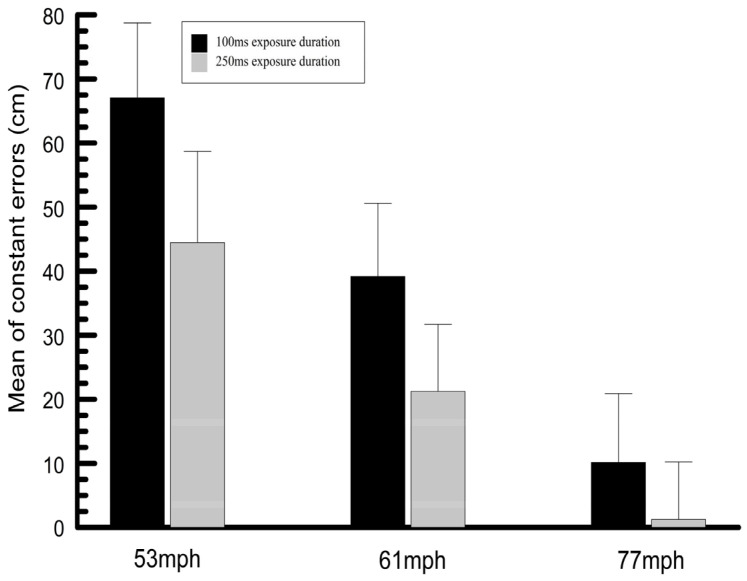
Mean (mean of all subject means) and standard deviation of the constant errors (passing height response—expected height response). Positive values indicate that the passing height responses were overestimated.

**Figure 8:**
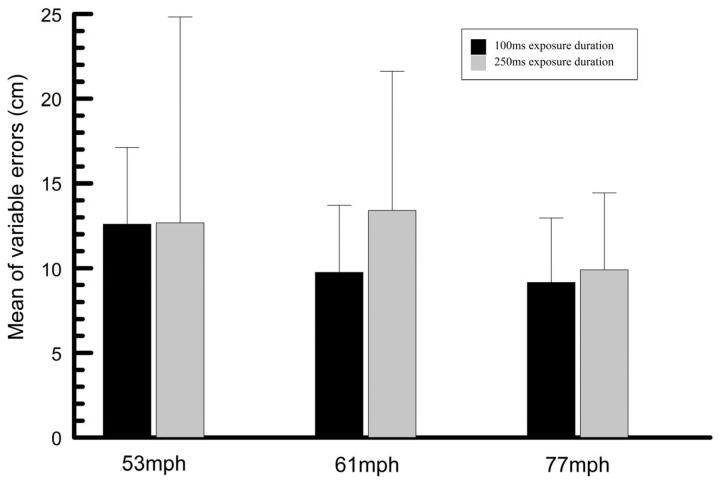
Mean (mean of all subject means) and standard deviation of the variable errors.

The mean difference between the passing height responses and the expected response (mean of measured heights) was determined for each combination of exposure duration and stimulus speed for all subjects. The means of these values for all subjects (mean of subject means), shown in Figure 7, are termed the mean constant errors. In addition, the mean of the standard deviations of these mean differences for each subject and the overall means of the standard deviations for all subjects combined (mean of subject means) were also calculated. These latter data are termed the variable errors and are plotted in Figure 8.

### 3.5. Calculations of Expected Passsing Height with Magnus and Drag Forces

A potential explanation for the overestimations of passing height found at the slower ball speeds (and at the faster ball speed for the 100 ms exposure duration) could be that subjects took into account the Magnus force or both the Magnus and drag forces. In order to assess whether these forces may have influenced the subjects’ responses, a trajectory calculator published by Alan Nathan: http://baseball.physics.illinois.edu/trajectory-calculator-old.html (accessed 18 December 2024) was used to calculate the passing height if the Magnus force and if the Magnus and drag forces were factored into the subjects’ responses. The passing height for a baseball, thrown from a distance of 40 ft (12.19 m) from the observer and from an initial height of 5.1 ft (1.55 m) from the ground, was determined using the trajectory calculator with the Magnus force and with both the Magnus and drag forces. The backspin revolution rate was set at 1500 rpm, representing a fastball. Pitches thrown by professional baseball players can reach much higher spin rates than those used here [33]. The Magnus force was applied in the trajectory calculator based on a model published by Hubbard [17].

The logic of performing two analyses (one with just the Magnus force and one with both the Magnus force and the drag force) was that if subjects accounted for the Magnus force, this would be consistent with the finding that subjects typically overestimated the ball’s height at most combinations of stimulus ball speed and exposure duration. The drag force may also be accounted for, but the drag force would only serve to modify the lift on the ball generated from the Magnus force. The drag force was considered a modifying variable because if subjects assumed that the drag force was the only force acting on the ball, then it would be expected that they would have underestimated the passing heights. Therefore, we did not calculate the passing heights that would have resulted if only the drag force was acting on the ball.

Drag forces on a tennis ball are likely to be different (perhaps higher) than those on a baseball due to the felt surface of a tennis ball and drag forces on tennis balls may vary from ball to ball because these forces decline as tennis balls age [34]. Further, differences in the circumference and weight of tennis balls and baseballs would most likely result in differences in the impact of the Magnus force on the trajectory of these balls. It is not possible to know whether subjects accounted for the fact that the balls used in this experiment were tennis balls. Therefore, the assumption in the following analyses is that subjects assumed that the pitched balls behaved as baseballs would. The results from the trajectory calculator when only the Magnus force and when both the Magnus and drag forces are included are shown in Table 3.

### 3.6. Summary of Results

Overall, the results demonstrate that while the mean passing height estimates were better at all speeds with the longer (250 ms) viewing duration than with the shorter (100 ms) viewing duration, the mean response was statistically different from the expected response at all combinations of viewing duration and ball speed except for the fastest speed and longer viewing duration combination. While the longer viewing duration improved predictions of passing height, it did not result in accurate responses for the medium and slow speeds. Passing heights were typically overestimated.

## 4. Discussion

The purpose of this study was to determine whether visual cues to estimate an approaching ball’s trajectory (including the linear speed of the ball, the rate of increase in the ball’s retinal image size, and the angular drop velocity of the ball) are reliable enough to estimate the passing height of the ball when situational and kinematic cues are limited and the ball’s launch angle is small and constant across ball speeds, and to determine whether a longer exposure duration improves passing height estimates compared to a shorter exposure duration.

### 4.1. Assessment of Ball-Flight Cues

There are several implications of the results. First, although the effect was modest, increasing the exposure duration did result in improved information content of the ball-flight cues related to passing height. In addition, the passing height estimates were not exactly as predicted from a simple linear extrapolation of the ball heights at 100 ms and 250 ms. One interpretation of these findings is that they provide evidence of the feasibility of online or prospective control in baseball batting, in which currently unfolding visual information plays a role in predicting the future location of the ball [3,35].

However, the deviation of passing height estimates from those predicted from a simple linear extrapolation may have been based on either visual information associated with ball-flight cues or an internal model that factored in gravity, so this deviation cannot be taken as evidence that subjects made use of ball-flight cues. Further, while the passing height estimates were improved with a longer exposure duration, these estimates were only accurate for one combination of ball speed and exposure duration (fastest ball speed and 250 ms exposure duration) and the variability of the passing height responses did not improve for the longer exposure durations. This could suggest the following. First, although the passing height responses did deviate somewhat from the responses expected from a linear extrapolation, this does not preclude the possibility that passing height estimates were primarily based on such an extrapolation. 

A second possibility for the poor passing height responses at the slower speeds is that visual cues such as time to collision variables or retinal angular velocities, which are included in models of passing height estimates described in the introduction, were subthreshold around the time the occluding spectacles were closed. In terms of time to collision, the threshold difference (Weber fraction) required to discriminate between rates of retinal image expansion is reported to be 8.5–14% [12]. The rates of retinal expansion were calculated as described in the methods, and then Weber fractions for the rate of retinal expansion were calculated for all comparisons of ball speed. The Weber fraction exceeded 8.5% at exposure durations of both 100 ms and 250 ms when comparing all ball speeds, although the influence of short exposure durations on discrimination thresholds for looming stimuli is not known and could have elevated the detection thresholds. In addition, other cues for time to collision or time to passage may have been available. For example, as mentioned before, the ball passed two vertical structural columns. Subjects may have used the time to traverse the distance to each column to help in determining the time to passage which could have lessened the need to accurately assess the rate of retinal expansion [11].

Calculations of the vertical retinal image velocities, also described in the methods, were used to generate Weber fractions for these velocities for all combinations of ball speed. These comparisons showed that at the exposure duration of 250 ms, the Weber fractions for the discrimination of retinal image velocity for all ball speeds was much greater than the threshold difference of 5% needed to discriminate angular velocities. At the exposure duration of 100 ms, the difference in retinal image velocity exceeded 5% when comparing the highest speed to the middle and low speeds. However, the difference in retinal image velocity between the two slower speeds did not exceed the 5% threshold [18,20,21].

Given that the aforementioned cues for passing height estimates are in most cases adequate for discriminating between the three ball speeds (ignoring the potential elevation of these thresholds at short exposure durations) for both exposure durations, the low accuracy and greater variability of the estimates of passing height at the two slower ball speeds raises a number of possibilities. First, although cues such as the rate of change in retinal image size and angular velocity could provide a basis for discriminating relative differences in passing height for the three ball speeds, these cues may not be adequate for absolute assessments of passing height. Alternatively, or in addition, it could be that while vertical retinal image velocity and the rate of retinal image expansion were adequate for estimating passing height, there may have been other variables that affect passing height estimates such as estimates of the elapsed time since the ball was released and the linear speed of the ball that are not processed accurately [18]. Evidence that poor speed discrimination may have contributed to the poor passing height estimates at the slower speeds was provided by Rushton and Duke, who demonstrated that speed discrimination for a flying object as it approaches is inaccurate [23]. On the other hand, a number of cues to linear speed were available in this experiment, including monocular (looming) and binocular cues, and the change in angle between the observer and the approaching object. Another cue for linear speed that might have been used was the different distances traversed for a given exposure duration for the different ball speeds (Figure 3), but as explained below, this cue relies on subjects being able to factor in the exposure duration. Perhaps, despite the availability of these cues, speed discrimination requires a greater viewing time of the ball than that allowed in this experiment. Any negative effects of the short exposure durations on passing height judgments may have been exacerbated in this experiment because, since subjects did not know which exposure duration would be used in a given trial, an estimate of the retinal image velocity and rate of change in retinal image size might have been made earlier in the ball’s trajectory than was necessary. This may have led subjects to misestimate the retinal image velocity and subsequently to misestimate the passing height of the ball.

There are at least three possible explanations, associated with limitations of ball-flight cues for estimates of passing height, which could account for the worse passing height judgments at the slower ball speeds compared to the faster speed for both exposure durations. First, if subjects did in fact rely on a linear extrapolation strategy, this could lead to better performance at the fastest speed (Figure 5). A second explanation for the worse performance at slower ball speeds is related to the launch angle. While the launch angle was deliberately maintained at a constant value at the three ball speeds to limit the contribution of this angle to passing height estimates, it may be that the combination of the slow speeds and the small launch angle was unfamiliar to subjects. In this case, subjects may have improperly evaluated the trajectory as a result of the combination of launch angle and ball speed at the slower ball speeds. A third reason for poorer passing height estimates at the slow speeds is as follows. In cases where the full trajectory of pitches is seen, a Bayesian model would generally predict that passing height estimates associated with ball-flight cues at slower ball speeds would be better than those at faster ball speeds. This is because with slower pitches, individuals would have more time to process ball-flight cues. However, in the current experiment, the same exposure durations were used for all ball speeds, which, for a given exposure duration, allowed for more of the trajectory of the pitch to be seen at the faster speed and which may have negated the usual advantage of a longer viewing time with slower pitches (Figure 3). The greater extent of the trajectory seen at the fastest speed may have contributed to the better passing height estimates at the fastest ball speed. However, for this distance cue to be used effectively, subjects would need to account for both the exposure duration and the ball speed. For example, the distance traveled by the slowest pitch over an exposure duration of 250 ms is greater than the distance traveled by the fastest pitch over a duration of 100 ms. In spite of this, passing height estimates were better for the fastest pitch at 100 ms compared to the slowest pitch at 250 ms. The potential distance cue probably also does not explain the better passing height estimates for the fast speed compared to the slower speeds at the 100 ms exposure duration, where the differences in the distance over which the ball was visible for the three ball speeds were relatively small. Further studies will be necessary to investigate whether subjects can factor in the exposure duration.

### 4.2. Cues Associated with Internal Models

Poor estimates of passing height might have resulted from limitations in assessing ball-flight cues for passing height estimates, perhaps exacerbated by the short exposure durations used in this study or the reliance on a sub-optimal linear extrapolation strategy. Another explanation for the poor judgments of passing height, and particularly for the overestimations of passing height at the two slower ball speeds, involves the use of internal models. Subjects may have based their passing height estimates on an internal model or prior distribution of ball trajectory that was heavily influenced by situational probabilities. If, for example, the observers assumed that the ball had underspin as would be the case with a typical fastball, then they may have predicted that the ball would drop less as a result of the Magnus force. This could have resulted in higher values for the estimated passing heights.

The passing height responses of subjects were compared to the calculated passing heights when the Magnus and drag forces were in effect. These comparisons are shown in Table 3. The results are modestly compelling, in that observers’ mean passing height estimates for some combinations of ball speed and duration were similar to the calculated passing heights if the Magnus and drag forces were in effect. It is known that rotational cues associated with the seams on a baseball can influence the perceived trajectory of the ball [14]. As described in the introduction, the direction of seam rotation (e.g., underspin or overspin) is related to the Magnus force. The white seams of the tennis balls used in the present study were low in contrast compared to the yellow-green color of the balls. Future investigations could repeat the occlusion studies used in this study with a ball that has higher contrast seams in order to provide the observer with a relatively direct indicator of the Magnus force. Such studies would help to determine whether assumptions about the Magnus and drag forces influence the passing height estimates.

Another situational probability that observers could have applied is the assumption that the ball would arrive in the strike zone [31]. While the strike zone is dependent on the subject’s height, if observers assumed that the ball would arrive in the strike zone, this could lead to overestimates of passing heights at the slower speeds. Measuring the height of each subject in future studies may allow for the testing of this hypothesis.

If observers do apply these situational probabilities to the passing height estimates, these assumptions may be quite strong. Evidence of the strength of assumptions based on situational probabilities comes from the fact that observers were shown nearly unobstructed examples of potential pitch trajectories prior to the data trials with the occlusion spectacles. While the observers were not told that these would in fact be two of the three pitch trajectories they would see in the experiment, they were at least aware of the potential for the balls to arrive at very low passing heights, thereby adding to the certainty of the prior information. Of course, it is possible that only two sample pitches were not enough to influence subsequent passing height estimates.

### 4.3. Limitations

The most significant potential limitation of this study is that the subjects’ responses were uncoupled. That is, the judged responses did not require the action necessary to bat a ball in an actual game. It is known that when perception and action are uncoupled, then pitch trajectory predictions could be negatively affected in some, but perhaps not all, cases compared to coupled situations [36,37]. Therefore, it is possible that had the participants in the current study been allowed to swing a bat at the approaching ball, height estimates, as assessed by the location of the bat, may have been more accurate than judgments of passing height. On the other hand, in a recent meta-analysis, it was suggested that cognitive tests of sports-related functions best discriminated between higher and lower skilled athletes when these tests used sports-specific task stimuli. However, no improvement in discriminating between athletes at different skill levels was gained by using sports-specific responses in cognitive tasks [38,39]. The current study made use of a sports-specific stimulus (i.e., the pitched balls), but did not require a sports-specific response (i.e., the bat swing). In addition, because of space limitations, it may be desirable to use uncoupled tasks for perceptual training in sports [40]. An understanding of potential differences in anticipatory performance on coupled and uncoupled tasks is necessary to determine whether perceptual training for sports performance is potentially effective under uncoupled conditions. Therefore, it will be important in a follow-up study to compare passing height estimates found in the current experiment with those in which a coupled task is required, such as batting or catching the approaching ball.

## 5. Conclusions

These data suggest that in the absence of explicit advance cues, visual ball-flight cues including the speed of the pitched ball and the angular retinal image velocity of the ball allow for better estimates of passing height at longer exposure durations. However, these ball-flight cues only allowed for accurate passing height judgments with the combination of longer exposure duration and faster ball speed. Passing heights were typically overestimated, particularly at slower ball speeds.

Potential conclusions are that ball-flight cues were inadequate for estimates of passing height, even at the longer exposure duration, or that the ball-flight cues were perceived to be unreliable because of the absence of earlier cues (e.g., kinematic cues) that could confirm that ball-flight cues were adequately reflective of the ball’s true trajectory. While it cannot be ruled out that subjects continued to rely on inadequate ball-flight cues or on a sub-optimal linear extrapolation strategy for passing height estimates under these conditions, only the linear extrapolation strategy would consistently result in overestimations of the passing heights unless, for example, the subjects routinely overestimated the ball speed for the two slower ball speeds. On the other hand, it is possible that subjects primarily based their passing height estimates on information associated with internal models of ball trajectory rather than on ball-flight cues. Calculations of the height of the balls when the Magnus force was in effect were more similar to the subjects’ passing height responses, at least at the 250 ms exposure duration (Table 3) compared to calculations based on linear extrapolation (Figure 5). This conception does not preclude the observer from switching between advance cues and ball-flight cues as the reliability of the ball-flight cues improves, as would be hypothesized in a Bayesian model. Such a cue-switching concept would also largely agree with the two-stage model of cricket batting developed by Müller and colleagues [41,42]. In this model, advance visual cues determine the position of the lower body and ball-flight information fine-tunes the bat swing. However, without the advance cues, observers may not demonstrate cue-switching.

## Figures and Tables

**Table 1:** Mean passing height responses and calculated (−1/2 gt^2^) responses.

	Exposure Duration	Mean Passing Height Response and Standard Deviation (and Standard Error) (cm From Ground)	Expected Range of Heights (cm Above the Ground) and Midpoint of the Measured Range (cm)	Calculated Height (cm) Using the Equation −1/2 gt^2^	Time for Ball to Traverse Total 40 ft Distance
Speed 1 (77 mph)	100 ms	109.23 ± 10.75 cm (2.40)	89.1–109.1 cm (99.1 cm)	90.9 cm	360.3 ± 7.41 ms
	250 ms	99.84 ± 8.83 cm (1.98)			
Speed 2 (61 mph)	100 ms	98.27 ± 11.41 cm (2.55)	50.7–67.5 cm (59.1 cm)	56.9 cm	446.9 ± 8.75 ms
	250 ms	79.89 ± 10.56 cm (2.36)			
Speed 3 (53 mph)	100 ms	88.04 ± 11.69 cm (2.61)	6.3–35.6 cm (21.0 cm)	22.9 cm	518.5 ± 14.9 ms
	250 ms	65.29 ± 14.58 cm (3.26)			

**Table 2:** Mean percentage of responses in the expected range for all subjects combined.

	Exposure Duration	Mean Percentage of Responses Within the Expected Range
Speed 1 (77 mph)	100 ms	31.0 ± 21.5%
	250 ms	54.0 ± 22.6%
Speed 2 (61 mph)	100 ms	4.0 ± 9.4%
	250 ms	18.0 ± 16.4%
Speed 3 (53 mph)	100 ms	0.5 ± 2.2%
	250 ms	1.5 ± 3.7%

**Table 3:** Experimental results simulated using a trajectory calculator (for a baseball) compared to the mean responses from subjects.

	Calculated Height (cm) Using the Equation −1/2 gt^2^	Calculated Height (cm) from Trajectory Calculator (Magnus Force (Top) and Magnus and drag Forces (Bottom))	Exposure Duration	Mean Height Response and Standard Deviation (and Standard Error)
Speed 1 (77 mph)	90.9 cm	116.62 cm 113.81 cm	100 ms	109.23 ± 10.75 cm (2.40)
			250 ms	99.84 ± 8.83 cm (1.98)
Speed 2 (61 mph)	56.9 cm	85.10 cm 80.74 cm	100 ms	98.27 ± 11.41 cm (2.55)
			250 ms	79.89 ± 10.56 cm (2.36)
Speed 3 (53 mph)	22.9 cm	54.35 cm 48.28 cm	100 ms	88.04 ± 11.69 cm (2.61)
			250 ms	65.29 ± 14.58 cm (3.26)

## Data Availability

The original contributions presented in the study are included in the article. Further inquiries can be directed to the corresponding author.

## Appendix A

**Table A1:** Individual mean and (and standard deviation) of passing height responses at each exposure duration and speed, and the percent correct responses (parentheses).

Subject	Speed 1 (77mph) Exposure 1 (100 ms) Exposure 2 (250 ms)	Speed 2 (61mph) Exposure 1 (100 ms) Exposure 2 (250 ms)	Speed 3 (53mph) Exposure 1 (100 ms) Exposure 2 (250 ms)
A	99.5 ± 11.17 (60) 93.2 ± 11.06 (50)	88.5 ± 14.54 (10) 74.5 ± 15.71 (30)	82.5 ± 12.96 (0) 61 ± 8.43 (0)
B	115.5 ± 7.25 (10) 106 ± 10.22 (50)	101.5 ± 8.51 (0) 85± 17.48 (10)	101 ± 7.75 (0) 61 ± 13.9 (0)
C	118.9 ± 9.47 (20) 107.7 ± 7.83 (60)	93.2 ± 14.97 (0) 82.9 ± 11.62 (10)	90.3 ± 10.25 (0) 52.1 ± 14.97 (0)
D	115.9 ± 6.19 (20) 100 ± 12.27(60)	102.6 ± 8.18 (0) 67.1 ± 14.93 (50)	87.4 ± 20.81 (0) 58.5 ± 12.39 (0)
E	114.6 ± 5.64 (20) 105.8 ± 4.71 (80)	108 ± 4.57 (0) 94.9 ± 5.45 (0)	106.7 ± 6.29 (0) 85.4 ± 5.66 (0)
F	116.2 ± 11.77 (30) 88.7 ± 8.82 (40)	89.7 ± 12.24 (0) 77.6 ± 9.11 (10)	75.4 ± 17.32 (0) 48.7 ± 11.09 (10)
G	119 ± 3.94 (0) 114.8 ± 5.9 (10)	98.5 ± 9.44 (0) 90 ± 13.94 (10)	78 ± 12.06 (0) 75 ± 12.69 (0)
H	84 ± 6.58 (40) 88 ± 4.83 (70)	74.5 ± 5.99 (10) 69 ± 10.22 (30)	71.5 ± 7.47 (0) 61 ± 5.68 (0)
I	109.5 ± 11.39 (50) 93.6 ± 10.06 (60)	101.7 ± 8.59 (0) 71.9 ± 8.03 (40)	89.8 ± 15.31 (0) 49.1 ± 9.57 (10)
J	117.1 ± 5.26 (10) 110.9 ± 8.18 (50)	114.7 ± 3.2 (0) 99.5 ± 4.65 (0)	104.1 ± 5.92 (0) 91.9 ± 7 (0)
K	108.8 ± 10.13 (40) 92.3 ± 8.53 (70)	93.9 ± 7.42 (0) 78.2 ± 10.46 (10)	74.7 ± 12.86 (0) 56.3 ± 8.01 (0)
L	106 ± 8.96 (50) 95 ± 8.3 (80)	102.3 ± 7.82 (0) 75.1 ± 7.08 (20)	93.8 ± 10.27 (0) 62.8 ± 6.89 (0)
M	100.6 ± 15.13 (50) 98.7 ± 6.31 (90)	80.1 ± 16.48 (30) 69.9 ± 12.18 (30)	80 ± 12.27 (0) 51.5 ± 7.11 (0)
N	96.2 ± 19.78 (10) 95.8 ± 9.16 (70)	98.7 ± 5.81 (0) 72.1 ± 15.62 (20)	80.7 ± 15.68 (0) 59.2 ± 12.37 (0)
O	102.2 ± 5.87 (80) 97 ± 10.36 (60)	96.2 ± 8.57 (0) 85.5 ± 9.13 (0)	93.4 ± 14.99 (0) 86.3 ± 7.38 (0)
P	120.2 ± 7.8 (20) 99.8 ± 10.26 (70)	107.9 ± 10.77 (0) 72.7 ± 14.14 (30)	91.3 ± 12.94 (0) 63.5 ± 13.44 (0)
Q	118.7 ± 8.58 (20) 109.5 ± 8.58 (50)	110 ± 10.65 (0) 84.8 ± 13.53 (10)	99.6 ± 9.11 (0) 75.3 ± 10.74 (0)
R	88.5 ± 12.7 (60) 84 ± 11.01 (30)	80 ± 17.48 (30) 70 ± 15.63 (50)	67 ± 16.02 (10) 43.5 ± 7.09 (10)
S	117.1 ± 5.8 (10) 113.9 ± 7.46 (10)	114.3 ± 6.88 (0) 103.5 ± 9.2 (0)	105.5 ± 9.14 (0) 90.1 ± 13.24 (0)
T	116 ± 9.66 (20) 102 ± 20.03 (20)	109 ± 12.87 (0) 73.5 ± 18.42 (0)	88 ± 22.39 (0) 73.5 ± 14.35 (0)
